# Superior Electromechanical Power at Rare‐Earth Manipulated Glassy Morphotropic Phase Transitions

**DOI:** 10.1002/advs.202415533

**Published:** 2025-06-04

**Authors:** Le Zhang, Liqiang He, Shuyuan Xu, Haoyu Wang, Yunlong Sun, Yating Ran, Dong Wang, Kaiyun Chen, Junkai Deng, Zibin Chen, Danyang Wang, Michael A. Carpenter, Sen Yang

**Affiliations:** ^1^ School of Physics and Frontier Institute of Science and Technology MOE Key Laboratory for Nonequilibrium Synthesis and Modulation of Condensed Matter and State Key Laboratory for Mechanical Behavior of Materials Xi'an Jiaotong University Xi'an 710049 China; ^2^ School of Materials Science and Engineering UNSW Sydney Sydney NSW 2052 Australia; ^3^ Department of Earth Sciences University of Cambridge Downing Street Cambridge CB2 3EQ UK; ^4^ State Key Laboratory of Ultra‐precision Machining Technology Department of Industrial and Systems Engineering The Hong Kong Polytechnic University Hong Kong 999077 China; ^5^ Research Institute for Advanced Manufacturing Department of Industrial and Systems Engineering The Hong Kong Polytechnic University Hong Kong 999077 China; ^6^ Advanced Materials Research Central Northwest Institute for Nonferrous Metal Research Xi'an 710016 China

**Keywords:** electromechanical power, glassy transition, morphotropic phase boundary, rare‐earth doping, thermal stability

## Abstract

High‐precision displacement control and the driving joints of artificial intelligence robotics, as well as advanced medical facilities, necessitate the use of superior lead‐free electromechanical materials that exhibit substantial electrostrain and driving force while maintaining thermal stability. In this study, an effective physical approach is developed to achieve significant enhancements of 135% and 50% in symmetric electrostrain and elastic modulus, respectively, of Sm‐doped (Bi,Na)TiO_3_‐BaTiO_3_ ceramics within the temperature range of 293–353K. This advancement facilitates a marked improvement in stress output. Unlike the prevalent focus on enhancing symmetric/asymmetric electrostrain output through polar coexistence states and defect dipoles, our approach induces superior electrostrain and stress power by inhibiting the formation of the R3c phase and manipulating the transition pathway in the morphotropic phase boundary composition of (Bi,Na)TiO_3_‐based ceramics via rare‐earth Sm doping. The achieved reversible glassy P4bm→P4mm and residual R3c→P4mm parallel transition paths, characterized by significant lattice expansion, enable the realization of potential electromechanical power across a broad temperature range. This approach overcomes the limitations of significant elastic softening and the deterioration of electrical properties with temperature variations at specific morphotropic/polymorphic phase transitions. Thus, it offers an effective method for generating superior electromechanical power in lead‐free ferroelectrics.

## Graphical abstract

An effective physical approach named Rare‐earth Manipulated Glassy Morphotropic Phase Transitions has been experimentally developed and theoretically validated to achieve significant enhancements of 135% and 50% in electrostrain and elastic modulus of lead‐free ferroelectrics respectively, further enabling the significant boost of requisite driving force in designed actuators.

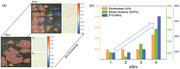

## 1 Introduction

In contemporary advanced manufacturing, particularly in the realms of 3D printing nanotechnology, high‐precision displacement control, and force driving in artificial intelligence robotics, as well as minimally invasive surgical facilities, there is a critical demand for functional materials that exhibit superior and precise electromechanical properties, such as substantial electrostrain and stress output with adequate thermal stability.^[^ ^1^, ^2^ ^]^ Perovskite ferroelectrics have emerged as promising candidates for these applications. Currently, the predominant commercial perovskite ferroelectrics remain lead‐based, such as Pb(Zr, Ti)O_3_ and Pb(Mg, Nb)O_3_‐PbTiO_3_ ceramics, due to their cost‐effectiveness and substantial piezoelectric constants across a broad temperature range.^[^ ^3^, ^4^, ^5^ ^]^ Despite significant global efforts by academic institutions and industries to design and fabricate high‐performance lead‐free piezoelectric systems,^[^ ^6^, ^7^, ^8^, ^9^ ^]^ aimed at overcoming regulatory restrictions on toxic lead in electronic devices, a critical challenge persists. Specifically, the question of how to achieve significant improvements in electrostrain and stress while maintaining excellent thermal stability in lead‐free piezoelectrics has remained unresolved for the past two decades. An effective approach for addressing this challenge would place lead‐free alternatives as indispensable components in high‐precision actuators and transducers.

The choice of phase convergence regions, such as the morphotropic phase boundary (MPB) between rhombohedral (R) and tetragonal (T) states in certain lead‐free piezoelectric ceramics—such as BaTiO_3_ and (K, Na)NbO_3_‐based systems—has successfully enhanced the piezoelectric constants *d_33_* to comparable levels to the commercial Pb(Zr, Ti)O_3_ ceramics at specific temperatures and compositions.^[^ ^6^, ^9^, ^10^ ^]^ However, these superior *d_33_* properties of lead‐free piezoelectric ceramics are confined to an extremely narrow temperature range. The issue of thermal stability in *d_33_* can be partially addressed through multilayered and textured ceramic fabrication methods.^[^ ^11^, ^12^ ^]^ Besides the typical lead‐free piezoelectric ceramics, some important molecular ferroelectrics such as *TMCM‐MnCl_3,_ (TMFM)_x_(TMCM)_1‐x_CdCl_3_ and HFPD* have attracted much attention in recent years and enlarged the research scope due to the promising *d*_33_, excellent flexibility, high biocompatibility.^[^ ^13^, ^14^, ^15^ ^]^ Despite various impressive studies achieving large asymmetric electrostrain by introducing defect dipoles,^[^ ^7^, ^16^, ^17^ ^]^ an effective physical approach for achieving large symmetric electrostrain output with excellent thermal stability in lead‐free systems remains elusive. The pronounced asymmetric electrostrain behavior significantly limits the design flexibility of electromechanical devices due to the necessity of considering the electric field direction. It is already revealed that relaxor‐to‐ferroelectric transitions of BNT‐based ceramics can achieve large symmetric electrostrains.^[^ ^18^, ^19^, ^20^, ^21^ ^]^ Recently, incorporating the MPB concept into relaxor‐to‐ferroelectric transitions of BNT ceramics can significantly decrease polar rotation energy, thereby further improving electrostrain outputs at the relaxor‐MPB (R3c/P4bm phases) transition point.^[^ ^22^ ^]^ Nevertheless, such large electrostrains still remain confined to a narrow transition temperature range. Traditional doping strategies for diffused lead‐free MPB or PPB regions have been tried in our group to enhance thermal stability of piezoelectric responses, but the electrostrain easily decreases or appears severe asymmetric effect due to the pinning effect on domain rotations and the hindrance of forming certain oriented ferroelectric states with large polarization and coupled spontaneous strain. Additionally, it is important to recognize that many previous studies have overlooked the significance of real drive force output when large electrostrain is obtained at specific phase transition regions. The usually chosen first‐order transitions often exhibit significant elastic softening, limiting the amplitude of stress/force output,^[^ ^23^, ^24^, ^25^ ^]^ which could pose challenges for actuator device design. The stress output determines the strength of the driving force, which is crucial in actuator and transducer applications, such as the muscles of intelligent robots and driving joints in advanced manufacturing instruments with heavy mass.

Recent findings have revealed that minimal amount of rare‐earth dopants, such as Samarium (Sm), can further reduce the energy profiles associated with ferroelectric rotations during MPB transitions.^[^ ^3^, ^4^ ^]^ This is attributed to the formation of structural heterogeneity, which holds significant promise for compensating the challenges associated with the hard rotation inherent in conventional doping methods.^[^ ^3^, ^4^ ^]^ Considering physical parallelism, this approach holds great prospects for boosting the ferroelectric and piezoelectric outputs at MPBs of the BNT systems. Moreover, the substitution of Sm^3+^ (with an ionic radius of ≈1.24 Å) at the A site (replacing Bi^3+^/Na^+^ with an ionic radius of ≈1.03 Å) is likely to induce strain heterogeneity in ferroelectrics.^[^ ^3^, ^26^ ^]^ This substitution may result in an optimized structural configuration that is both glassy and stable across a wide temperature range.^[^ ^27^, ^28^, ^29^, ^30^ ^]^ It is reasonably anticipated that incorporating an appropriate amount of rare‐earth dopants into an MPB composition can lead to large electrostrain with excellent thermal stability. Additionally, the influence of rare‐earth doping on the elastic modulus warrants further investigation to elucidate its impact on stress output capability.

For the present study, a ceramic system exhibiting large electrostrain and a hardened elastic modulus with thermal stability has been successfully developed by doping rare‐earth Sm into a 0.01Mn‐0.94(Bi, Na)TiO_3_‐0.06BaTiO_3_ matrix (with Sm concentrations of 0.1, 0.2, 0.3, and 0.4 mol%, denoted as 1, 2, 3, and 4Sm, respectively). This system undergoes morphotropic phase transitions below the temperature *T*_d_ of relaxor‐ferroelectric transitions. Notably, the 4Sm ceramics achieve simultaneous enhancements of 135% in electrostrain and 50% in elastic modulus when compared to neighboring compositions, thereby enabling a significant increase in stress output. The glassy state persists over a wide temperature range, facilitating the occurrence of a large symmetric electrostrain of ≈0.47% and a modulus of ≈135 GPa within the temperature range of 293–353 K, which meets the requirements of most electromechanical devices. Density functional theory (DFT) and phase field simulation results indicate that the superior electromechanical performance achieved through Sm doping is attributed to the inhibition of R3c phase formation and the reversal of the original field‐induced P4bm→P4mm→R3c transition sequence at the MPB region to reversible P4bm→P4 mm and R3c→P4 mm simultaneous glassy transitions.

## 2 Experimental Results and Analysis

**Figure 1:**
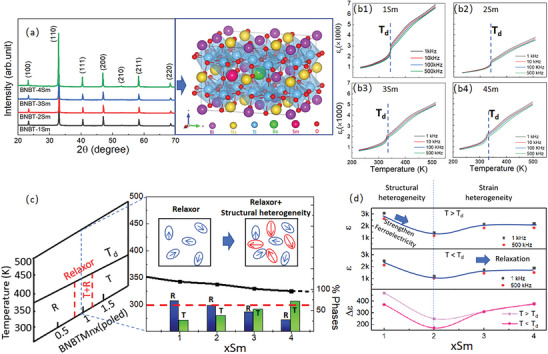
a) X‐ray diffraction patterns of poled BNBT‐1, 2, 3, and 4Sm ceramics at 300 K in the 2θ range of 20–70°, indexed on the basis of BNBT perovskite structure, the microstructure of BNBT‐xSm lattice is given in the right set. b1‐b4) Dielectric permittivity versus temperature curves of poled 1‐4 Sm ceramics measured in a frequency range of 1–500 kHz. Poling of the ceramics was carried out under a DC electric field of ≈55 kV cm^−1^ at 300 K. c) Phase diagram of BNT‐BT matrix with a MPB and the relation with the BNBT‐xSm phase diagram displaying microstructure evolution and phase transition information. d) Dielectric permittivity values under 1 and 500 kHz at the temperatures slightly above/below T_d_ and the composition dependent ∆ɛ.

**Figure** **1**a presents the X‐ray diffraction (XRD) data for the 1‐4 Sm ceramics, confirming the presence of perovskite structures without any impurities. The refinement analysis of XRD data (refer to Figure S1; Table S1, Supporting Information) illustrates the fluctuations of the rhombohedral (R3c) and tetragonal (P4bm) phases at room temperature. The synthesis process of the ceramic samples and the characterization methods employed are described in the experimental and calculation section of supportive materials. The actual compositions of 1‐4 Sm are given in Figure S2 (Supporting Information). Figure 1(b1‐b4) displays the temperature dependence of the dielectric permittivity (*ε_r_*) for the 1‐4 Sm ceramics, measured at frequencies of 1, 10, 100, and 500 kHz over the temperature range of 220–500 K. Prior to the dielectric testing, all ceramic samples were poled under a electric field of ≈55 kV cm^−1^ at room temperature to clearly delineate the relaxor‐ferroelectric transition temperature *T*_d_. It is observed that *T*_d_ value decreases slightly from 350 to 330 K as the concentration of Sm dopants increases from 1Sm to 4Sm.

Figure 1c illustrates the phase diagram of the xSm ceramic system, based on XRD and dielectric analyses. It is evident that the proportion of the T phase increases with the addition of Sm in the BNT‐BT ceramics, highlighting Sm's role in stabilizing the domain state with T symmetry. Figure 1d depicts the frequency dependence of dielectric constants above and below *T*_d_ for the 1‐4Sm compositions. The dielectric constants at *T*>*T*_d_ and *T*<*T*_d_ initially decrease from 1 to 2Sm and then increase from 2 to 4Sm, resulting in a dip at the 2 Sm composition. This dip in dielectric constants is attributed to the enhanced ferroelectricity from 1 to 2Sm, due to the development of local structural heterogeneity into both relaxor and ferroelectric states.^[^ ^3^, ^4^ ^]^ The slight increase in dielectric constants from 2 to 4Sm is attributed to the emergence of more flexible nanodomain patterns, resulting from the additional strain heterogeneity caused by the Sm^3+^ (with an ionic radius of ≈1.24 Å) substituting for Bi^3+^ (with an average ionic radius of ≈1.02 Å).^[^ ^4^, ^23^, ^26^ ^]^ Furthermore, this strain heterogeneity accounts for the decreased value of *T*_d_ and causes the sharp decline in dielectric permittivity near *T*_d_ to become gradually weakened and diffused with varying test frequency. The degree of frequency dependence near *T*_d_ is characterized by the difference in dielectric constants Δ*ε* at 1 and 500 kHz. The 3‐4Sm compositions exhibit nearly identical Δ*ε* values above and below *T*_d_, in contrast to the Δ*ε* divergence observed above and below *T*_d_ in the 1‐2Sm samples.

**Figure 2:**
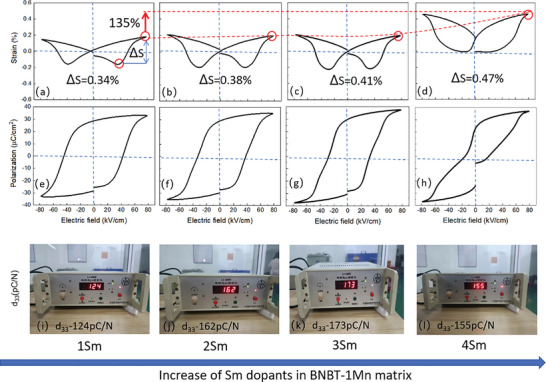
a–d)P‐E loops and e–h) S‐E curves i–l) *d*_33_ values of 1, 2, 3, and 4 Sm ceramics respectively at room temperature measured under 80 kV cm^−1^ and 1 Hz. ∆S, difference of positive and negative electrostrain as indicated by the blue arrow, represents the maximum potential electrostrain outputs arising from polarization rotation from disordered polar orientation to ordered one.

**Figure** **2** presents the room‐temperature polarization‐electric field (P‐E) and strain‐electric field (S‐E) loops for the 1‐4 Sm ceramics under a moderate electric field of 80 kV cm^−1^ at a frequency of 1 Hz. The test temperature, indicated by the dashed red line in Figure 1c, is significantly distant from the *T*_d_ line, suggesting that the observed superior electrical properties are not associated with typical relaxor‐ferroelectric transitions. The difference in electrostrain (Δ*S*), defined as the difference between positive and negative electrostrain, increases from 0.34% in 1Sm to 0.47% in 4Sm, indicating an ≈40% increase in the maximum potential electrostrain output as the system transitions from a disordered polar state to an ordered polar state in the MPB state following Sm doping, as shown in Figure 2a–d. This enhancement is strongly correlated with the strengthened ferroelectricity and strain‐polar coupling induced by Sm rare‐earth doping. The increase of negative electrostrain from 1 to 3Sm is ascribed to the increase of remnant polarization *P*_r_. The maximum of negative strain at 3Sm is connected to the disordering process of largest remnant polarization to the zero polarization. The composition dependence of *P*_m_, *P*_r_, *E*_c_ is given in Figure S3 (Supporting Information).

Notably, the negative electrostrain nearly vanishes in the 4Sm composition, resulting in a 135% increase in applicable electrostrain output compared to the 1–3Sm compositions. The P‐E and S‐E loops from 1 to 4 Sm gradually become narrower (Figure 2a–h), with the 4Sm composition exhibiting an abnormal girdled shape and a significant decrease in the coercive field (*E*_c_). The reduced *E*_c_ with Sm doping indicates easier rotation of ferroelectric domains, which is advantageous for optimizing piezoelectric responses. The balance between the lowered *E*_c_ and increased remnant/saturation polarizations from 1 to 3Sm results in the largest piezoelectric coefficient *d*_33_ of ≈174 pC/N at 3Sm, representing a 40% increase compared to 1Sm, as shown in Figure 2i–l). The 4Sm sample has a slight decrease in *d*_33_ due to a minor reduction in remnant polarization compared to 3Sm. Based on XRD analysis, the improvement in *d*_33_ is likely related to the well‐mixing of nearly equal proportion of R and T phases.

**Figure 3:**
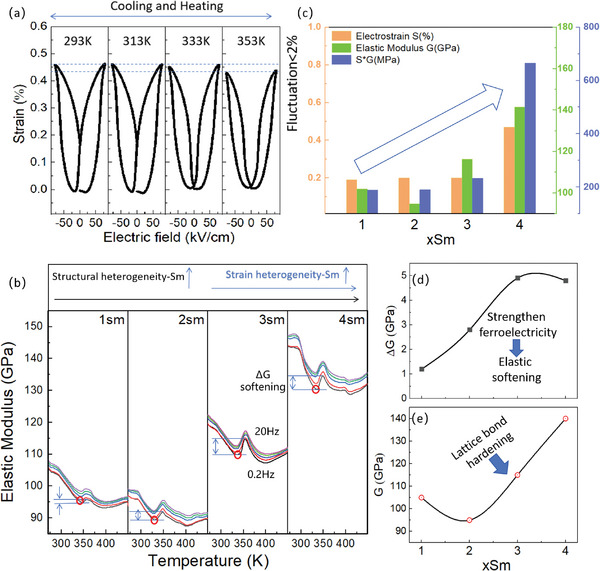
a)Temperature dependence of electrostrain of 4Sm ceramics in the temperature range of 293–353K, b) Temperature dependent elastic modulus curves of 1‐4Sm ceramics within the temperature range of 273‐450 K under driving frequencies 0.2–20 Hz, c)The composition dependence of electrostrain, elastic modulus and S×G(stress) at room temperature, d) Elastic softening ∆G and e) elastic modulus G slightly below T_d_ at 1 Hz, a frequency setting chosen to be consistent with the test frequency of P‐E and S‐E loops.

**Figure** **3**a illustrates that the large electrostrain of ≈0.47% in the 4Sm composition is well‐maintained within the temperature range of 293–353 K, exhibiting only slight fluctuations of less than 2%, even when crossing the *T*_d_ temperature. Figure 3b demonstrates the temperature‐dependent elastic modulus of poled 1‐4Sm samples over the temperature range 273–500 K when measured at frequencies of 0.2–20 Hz. The presence of more Sm cations (3‐4Sm) significantly hardens the elastic modulus (*G*) of the matrix, which is advantageous for enhancing stress/force output, estimated here by the product *S*×*G*. A comparison of room‐temperature electrostrain (*S*), elastic modulus *G*, and estimated stress (*S*×*G*) in the xSm system is summarized in Figure 3c. The combination of thermally stable electrostrain and large modulus in the 4 Sm composition enables a 3–3.5‐fold increase in *S*×*G* compared to compositions with lower Sm doping levels, such as 1Sm. Although elastic softening is observed near the *T*_d_ temperatures of each poled xSm ceramic, and the degree of softening (Δ*G* at 1 Hz) intensifies with increased Sm content, as shown in Figure 3d, the absolute elastic modulus level of 1‐4Sm within the temperature range of 290–400 K generally increases from 90–100 GPa in 1–2Sm to 130–140 GPa in 4Sm, as depicted in Figure 3e.

## 3 Simulation Results and Discussion

It is well established that enhancing electrostrain through the use of simple phase transitions,^[^ ^31^, ^32^ ^]^ such as morphotropic and polymorphic phase boundaries, is typically accompanied by significant elastic softening,^[^ ^20^, ^21^, ^22^ ^]^ which greatly restricts the stress/force output for displacement actuation. Our current findings demonstrate that the conventional relationship between large strain and low elastic modulus in ferroelectrics can be overcome by manipulating the structural configuration of R and T phases with a trace amount of rare‐earth Sm dopants. The experimental results suggest that this superior electromechanical performance, coupled with good thermal stability, may be linked to the instability of the R phase and the balance between structural and strain heterogeneity. However, a comprehensive understanding of this advantageous trade‐off between strain and modulus remains elusive, primarily due to a lack of insight into the dynamic transition processes driven by an electric field.

Given the significant challenges associated with determining the field‐and temperature‐dependent dynamic processes of short‐range ordered relaxor states in the BNT system using *in‐situ* HRTEM techniques, multi‐scale simulation methods have been employed to elucidate the physical mechanisms underlying these intriguing phenomena. These simulations provide clear guidance on achieving superior electromechanical performance in lead‐free BNT ferroelectrics, moving beyond the conventional approach of constructing R/T nanodomains in an ambiguous manner. The simulations aim to address two critical questions: 1) What are the unique characteristics of microstructure evolution and phase transitions in Sm‐modified BNT‐BT MPB compositions that contribute to superior electromechanical performance, specifically in terms of electrostrain and modulus? and 2) Why can this electromechanical performance be maintained across a wide temperature range? By answering these questions, the simulations offer insights into the design and optimization of lead‐free BNT ferroelectrics for advanced electromechanical applications.

**Figure 4:**
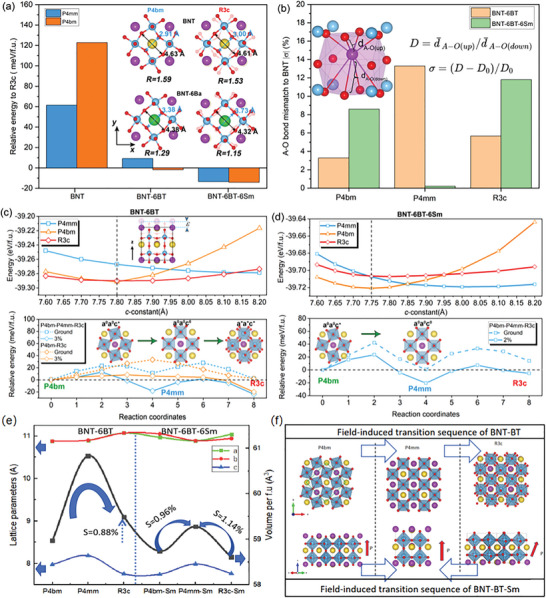
Phase stability and transition barriers among P4bm, P4mm, and R3c with Ba and Sm doping into BNT. a) The relative energy of P4bm and P4mm phases with respect to R3c phase (set to zero) in BNT, Ba and Ba‐Sm co‐doped BNT. The inset plots show the shorter and larger O‐O distance at the A site of P4bm and R3c phases with and without Ba doping. R, the ratio between larger and shorter O‐O distance, is adopted to quantify the local rotation degree at A site. b) Local lattice distortion of P4bm, P4mm, and R3c phases of BNT‐6BT and BNT‐6BT‐6Sm relative to the A‐site of BNT (see detailed description in calculation methods part of supporting information). c,d) Energy changes and energy barriers between P4bm, P4mm, and R3c states of BNT‐6BT and BNT‐6BT‐6Sm under different *z*‐direction tensile strains with varying lattice parameter c. e) The variations of lattice parameters *a*, *b*, *c* and Volume of BNT‐6BT and BNT‐6BT‐6Sm super lattice, f) The summary for transition route changes in BNT‐6BT before and after Sm doping.

To make clear the modulated phase stability and transitions by Ba and Sm doping, DFT calculations were conducted. **Figure** **4**a presents the relative energy (measured in meV per ABO_3_ formula unit) of the P4bm and P4mm phases with respect to the R3c state in BNT, 6% Ba‐doped BNT (BNT‐6BT), and 6% Ba‐6% Sm co‐doped BNT (BNT‐6BT‐6Sm) systems, as determined by the DFT method (details in the DFT simulation section of supportive materials).

Upon doping 6% Barium cations into the A‐site of the BNT matrix (Ba^2+^ replacing Na^+^ cations, which displays the lowest energy), the relative energy levels of P4mm (ΔE*_P_*_4_ *_bm_* = 9.16 meV/f.u) and P4bm (ΔE*_P_*_4_ *_mm_* = 1.89 meV/f.u.) are both close to that of R3c. This suggests the potential coexistence of R3c, P4bm, and P4mm states in BNT‐6BT, differing from the reported stable R3c phase in pure BNT and aligning with previous findings in BNT‐BT system.^[^ ^33^, ^34^ ^]^ The reduced energy difference between R3c, P4bm, and P4 mm after Ba doping is attributed to the decreased tilt angle and octahedral distortion, influenced by the strain heterogeneity during the substitution process of Ba (r_Ba2+_≈1.35 Å) for Na (r_Na+_≈1.02 Å), as illustrated by the lattice structure changes in the inset images of Figure 4a. Reduction of tilt angle and octahedral distortion counteract the increase in local elastic energy associated with Ba doping in lattice, thereby enhancing the system's thermodynamic stability. The reduction in the O‐O bond ratio *R* in both P4bm and R3c phases, indicated by the division of length of black arrow to that of blue arrow, gives the evidence for that Ba at the A‐site suppresses Ti‐O octahedral rotation. The smaller decrease in the O‐O ratio in the P4bm phase compared to the R3c phase indicates that P4bm is more adaptable to Ba. Such a phase evolution with Ba doping in simulation mode is well consistent with experimental results and provides robust verification of the reliability of our DFT calculations.

The influence of Sm on the phase stability and transition pathway of BNT‐6BT under an electric field has been further investigated through calculations. Sm doping makes the P4bm phase become the most stable structure, as opposed to the R3c phase. Additionally, the energy of the P4bm phase is very close to that of the P4 mm phase, with a minimal energy difference of ≈0.72 meV per formula unit, as shown on the right side of Figure 4a. Consequently, the microstructural pattern under Sm doping is likely to favor the formation of the T phase with both P4bm and P4mm symmetries, leading to a reduced percentage of the R3c phase. The increased T phase percentage in BNT‐6BT following Sm doping is attributed to the reduction in the A‐O bond mismatch degree, *σ* = (*D*−*D_0_*)/*D_0_*, of the BNT‐BT matrix, where D = *d_up_*/*d_down_* and *D*_0_ is the *D* of the BNT matrix (see details in the computational methods section). The *σ* values in the Ba‐polyhedra of the P4bm, P4mm, and R3c phases in BNT‐BT are initially presented in the yellow columns of Figure 4b. Upon Sm doping into BNT‐BT to replace Bi, the *σ* value of the P4mm phase significantly decreases, indicating the smallest mismatch with the Bi‐polyhedra. In other words, the P4mm structure is more compatible with Sm, allowing the largest energy changes of P4mm after Sm doping compared with the P4bm case (refer to Figure 4a). Conversely, the Sm site exhibits the largest mismatch in the R3c phase, substantially increasing the instability of the R phase. In comparison with A‐O bond mismatch, the B‐O bond mismatch can not reflect the symmetry and the structure character changes in R3c and P4bm states before and after Sm doping in BNT‐BT system because the Sm and Ba are both entering the A‐site of BNT matrix.

The energy profiles of the R3c, P4bm, and P4mm phases as functions of *z*‐axis strain have also been calculated for both Ba‐doped and Ba‐Sm‐doped BNT lattice structures to investigate the characteristics of the phase transition path under an electric field (*E*), as depicted at the top of Figs. 4(c) and 4(d), respectively. The zero strain states of stablest P4bm along the *z*‐axis located at *c* lattice parameter amounting to 7.75−7.8 Å, due to the lowest total energy after relaxation, as indicated by the black dashed lines. The increase of c‐axis constants will generate larger strain changes for P4bm rather than the P4 mm cases due to the smaller *c*‐lattice constant of initial P4bm phase. It significantly promotes the free energy of P4bm in both BNT‐BT and BNT‐BT‐Sm lattices and push forward the transition to P4 mm states with larger *c*‐constants and the suppression of polar alignment in *a‐b* plane. In the case of Ba‐doped BNT, the phase transition generally follows the P4bm/P4mm→R3c path, as R3c remains the most stable phase when a significant *z*‐axis strain is applied in the range of 0–3.85% (*c*:7.8−8.1 Å). To examine the kinetic transition process in 6% Ba‐doped BNT, the climbing image nudged elastic band (Cl‐NEB) method was employed to calculate the phase transition barriers for the two possible paths in terms of energy: the P4bm‐to‐R3c and P4bm‐to‐P4mm‐to‐R3c processes. As shown at the bottom of Figure 4c, the energy barrier for the transition from P4bm to R3c (orange dotted line) is ≈33.24 meV per f.u., which is larger than the barrier for the transition from P4bm to P4mm (blue dotted line), ≈22.82 meV per f.u. This suggests that P4bm is likely to transition to P4mm under a small electric field. Under a large electric field (≈3% *c*‐axis strain), both P4bm and P4mm are expected to convert to R3c due to the phase stability of the R phase, consistent with previous experimental results indicating a field‐induced P4bm→P4mm→R3c transition.^[^ ^33^ ^]^

In the Ba/Sm co‐doped BNT system, *z*‐axial strain influences the energy inversion between the P4bm and P4mm phases. Specifically, P4bm becomes more stable than P4mm when the lattice parameter *c* is less than 7.85 Å, whereas P4mm becomes the stable phase when c exceeds 7.85 Å, as illustrated at the top of Figure 4d. In contrast, the energy of the R3c phase remains higher than that of P4mm when *c* is less than 7.95 Å (strain:2.58%). This indicates that Sm dopants enhance the proportion of P4bm‐P4mm transitions in the BNT‐BT system while diminishing the contributions from the R3c phase when strain < 2.58%. The bottom of Figure 4d presents the CI‐NEB results for zero and 2% *z*‐axial strain (with *c* = 7.9 Å) along the P4bm‐P4mm transition path. When a 2% strain is applied to the 6% Sm‐doped lattice, the R3c and P4bm phases become unstable when compared to P4mm, altering the transition path to P4bm‐P4mm and resident R3c‐P4mm transitions. This differs from the electric field‐induced P4bm‐to‐P4mm‐to‐R3c transition sequence previously reported in BNT‐BT. The transition barriers for P4bm‐to‐P4mm and R3c‐to‐P4mm (indicated by the solid blue line) further decrease to 23.64 meV per formula unit and ≈4.9 meV per formula unit, respectively. Figure 4e,f depict the variations in unit cell structure, dimensions, and volume for the P4bm, P4mm, and R3c phases in BNT‐6BT and BNT‐6BT‐6%Sm. These results suggest that the P4bm‐P4mm transition in BNT‐6BT is associated with large electrostrain, but P4mm may transform to R3c under a large electric field, potentially limiting the electrostrain amplitude at the MPB. Sm doping hinders the onset of the R3c phase and strengthens the P4bm‐P4mm and R3c‐P4mm transitions. The large electrostrain observed in 4Sm is attributed to the inhibited formation of the R3c phase and the reversal of P4mm→R3c transitions, thereby promoting the formation of more T phase. Although the lattice parameters *a*, *b*, and *c* of the three phases slightly decrease with Sm doping, the strain output from volume changes during transitions remains unaffected. The estimated strain output from the field‐induced P4bm‐to‐R3c transition route through P4mm in BNT‐BT is lower than those of both P4bm→P4 mm and R3c→P4mm transitions. Furthermore, the decreased *a*, *b*, and *c* parameters strengthen the A‐O/B‐O bonds, playing a crucial role in increasing the elastic modulus. The decreasing trend of a, b, and c observed in DFT simulations is corroborated by the variation of lattice parameters in the 1‐4Sm samples, as shown in Table S1 (Supporting Information).

**Figure 5:**
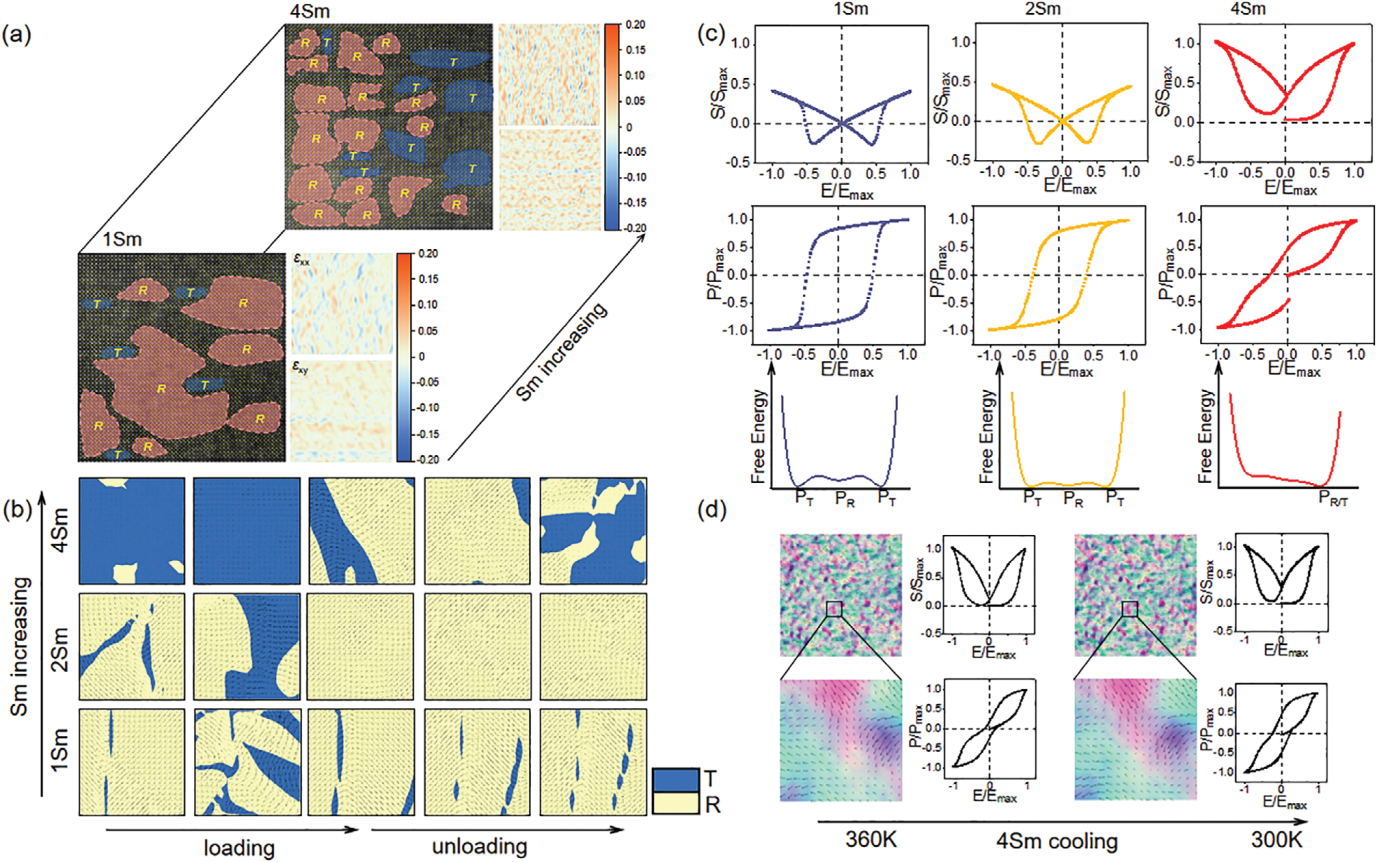
a) HAADF‐TEM and GPA pattern obtained from different regions of one grain in poled 1 and 4Sm ceramics along [001] incidence, showing R+T coexistence and phase fluctuation with increasing Sm. b,c) Phase field simulation results for the evolution of microstructure, properties, and Landau energy profiles with increase of electric field (E) and Sm doping level, respectively. d)The temperature dependence of microstructure and associate P‐E /S‐E loops in 4Sm from 360 to 300 K.

**Figure** **5**a illustrates the evolution of polarization and coupled lattice strain from 1Sm to 4Sm, utilizing high‐angle annular dark‐field transmission electron microscopy (HAADF‐TEM) and geometric phase analysis (GPA) experimental techniques. The morphological images are captured from different regions within a single grain along the [001] incidence direction. In the 1Sm composition, a domain state with more R and fewer T phases is observed. As the Sm content increases to 4Sm, a greater presence of the T phase and a more diffused distribution of the R/T domain pattern emerge compared to the 1Sm composition. This indicates that Sm doping induces a transformation of some R phase regions into the T phase, further corroborating the DFT results. Additionally, the diffused glassy process of the R/T ferroelectric state, arising from strain heterogeneity, is also observed. Phase field simulations are provided on a macro scale to demonstrate how rare‐earth Sm dopants influence the percentage and domain configuration of the glassy R/T state, thereby affecting overall electrostrain output and thermal stability. The control of Sm composition in these simulations is more precise and closely aligned with the nominal doping level than in DFT simulations. The smallest Sm dopant level in DFT simulations is set at 6% due to the limitations of the DFT simulation scale. Detailed phase field simulation methods are described in the supporting information.

The distinct tensor of lattice strain (*ε_xx_* and *ε_xy_*) for 1Sm and 4Sm, as depicted in Figure 5a, is incorporated into phase‐field modeling to simulate the actual microstructural evolution with varying Sm composition following one cycle of electric field application and removal (poling process) in the MPB region of BNT‐BT below *T*_d_, as shown in Figure 5b. The percentage of the T phase at *E* = 0 clearly increases with higher Sm doping. For the low Sm doping level (1Sm) at the bottom of Figure 5b, it is evident that the T phase predominantly transitions to the R phase state upon the application of an electric field, as the same as the DFT anticipation. With a slight increase in Sm content, the entire T phase can transition to the R phase state under the electric field due to the reduced energy barrier of the MPB facilitated by structural heterogeneity. As Sm content is further increased, the R phase becomes progressively hindered, and more T phase regions recovered after the removal of the electric field, indicating an improvement in the reversibility of the electrostrain.

The associated P‐E and S‐E loops are presented at the top of Figure 5c, demonstrating strong consistency with the experimental results. The bottom of Figure 5c illustrates the evolution of the corresponding Landau energy profiles (at *E* = 0) with increasing Sm content. The energy profiles of the R‐T transition at the MPB become flattened at the onset of Sm doping due to the formation of structural heterogeneity. This flattening is the primary reason for the variations in room‐temperature *d_33_* and polarization observed with lower Sm compositions. The initial R/T states can be stabilized into more stable glassy configurations with lower energy at higher Sm doping levels, owing to the stronger influence on the phase transitions. Additionally, the thermally stable glassy domain patterns in high Sm doping facilitate the development of specific microstructures that enable superior electrical properties over a wide temperature range, as depicted in Figure 5d.

## 4 Overlook

In summary, DFT and phase field simulations indicate that significant enhancements in electromechanical performance—specifically, large electrostrain and elastic modulus with thermal stability can be achieved in the relaxor‐glassy MPB composition of (Bi,Na)TiO_3_‐based ceramics through rare‐earth Sm doping, which impedes the formation of the R3c phase. Consequently, the electrostrain and estimated stress are improved by 135% and 3‐3.5 times, respectively, compared to neighboring compositions, and these enhancements can be maintained within the temperature range of 290–353 K, largely independent of the relaxor‐ferroelectric transition at 330 K. Our strategy offers an effective approach for achieving substantial electrostrain outputs and a desirable large modulus with adequate thermal stability, thereby meeting the application requirements for actuators and transducer devices in intelligent robots and advanced manufacturing instruments with heavy mass.

## Conflict of Interest

The authors declare no conflict of interest.

## Supporting information



Supporting Information

## Data Availability

The data that support the findings of this study are available from the corresponding author upon reasonable request.

## References

[1] MarketsandMarkets , Piezoelectric Devices Market, 2021.

[2] K. Uchino , Advanced Piezoelectric Materials: Science and Technology, Woodhead Publishing, Cambridgeshire UK 2017.

[3] F. Li , M. J. Cabral , B. Xu , Z. Cheng , E. C. Dickey , J. M. LeBeau , J. Wang , J. Luo , S. Taylor , W. Hackenberger , L. Bellaiche , Z. Xu , L.‐Q. Chen , T. R. Shrout , S. Zhang , Science 2019, 364, 264. 31000659 10.1126/science.aaw2781

[4] F. Li , D. Lin , Z. Chen , Z. Cheng , J. Wang , C. Li , Z. Xu , Q. Huang , X. Liao , L.‐Q. Chen , T. R. Shrout , S. Zhang , Nat. Mater. 2018, 17, 349. 29555999 10.1038/s41563-018-0034-4

[5] B. Jaffe , W. R. Cook , H. Jaffe , Piezoelectric Ceramics, Academic Press, New York 1971.

[6] K. Xu , J. Li , X. Lv , J. Wu , X. Zhang , D. Xiao , J. Zhu , Adv. Mater. 2016, 28, 8519. 27441456 10.1002/adma.201601859

[7] X. Liu , X. Tan , Adv. Mater. 2016, 28, 574. 26596685 10.1002/adma.201503768

[8] X. Wang , J. Wu , D. Xiao , J. Zhu , X. Cheng , T. Zheng , B. Zhang , X. Lou , X. Wang , J. Am. Chem. Soc. 2014, 136, 2905. 24499419 10.1021/ja500076h

[9] W. Liu , X. Ren , Phys. Rev. Lett. 2009, 103, 257602. 20366285 10.1103/PhysRevLett.103.257602

[10] Q. Zhang , Y. Zhang , F. Wang , Y. Wang , D. Lin , X. Zhao , H. Luo , W. Ge , D. Viehland , Appl. Phys. Lett. 2009, 95, 102904.

[11] T. Zheng , Y. Yu , H. Lei , F. Li , S. Zhang , J. Zhu , J. Wu , Adv. Mater. 2022, 34, 2109175. 10.1002/adma.202109175 34907605

[12] L. Xu , J. Lin , Y. Yang , Z. Zhao , X. Shi , G. Ge , J. Qian , C. Shi , G. Li , S. Wang , Y. Zhang , P. Li , B. Shen , Z. Fu , H. Wu , H. Huang , F. Li , X. Ding , J. Sun , J. Zhai , Nat. Commun. 2024, 15, 9018. 39424820 10.1038/s41467-024-53437-5 PMC11489714

[13] Y.‐M. You , W.‐Q. Liao , D. Zhao , H.‐Y. Ye , Y. Zhang , Q. Zhou , X. Niu , J. Wang , P.‐F. Li , D.‐W. Fu , Z. Wang , S. Gao , K. Yang , J.‐M. Liu , J. Li , Y. Yan , R.‐G. Xiong , Science 2017, 357, 306. 28729511 10.1126/science.aai8535

[14] W.‐Q. Liao , D. Zhao , Y.‐Y. Tang , Y. Zhang , P.‐F. Li , P.‐P. Shi , X.‐G. Chen , Y.‐M. You , R.‐G. Xiong , Science 2019, 363, 1206. 30872522 10.1126/science.aav3057

[15] H.‐Y. Zhang , Y.‐Y. Tang , Z.‐X. Gu , P. Wang , X.‐G. Chen , H.‐P. Lv , P.‐F. Li , Q. Jiang , N. Gu , S. Ren , R.‐G. Xiong , Science 2024, 383, 1492. 38547269 10.1126/science.adj1946

[16] H. Luo , H. Liu , H. Huang , Y. Song , M. G. Tucker , Z. Sun , Y. Yao , B. Gao , Y. Ren , M. Tang , Sci. Adv. 2023, 9, eade7078. 36735779 10.1126/sciadv.ade7078 PMC9897659

[17] G. Huangfu , K. Zeng , B. Wang , J. Wang , Z. Fu , F. Xu , S. Zhang , H. Luo , D. Viehland , Y. Guo , Science 2022, 378, 1125. 36480626 10.1126/science.ade2964

[18] S.‐T. Zhang , A. B. Kounga , E. Aulbach , H. Ehrenberg , J. Rödel, Appl. Phys. Lett. 2007, 91, 112906.

[19] T. Li , X. Lou , X. Ke , S. Cheng , S. Mi , X. Wang , J. Shi , X. Liu , G. Dong , H. Fan , Y. Wang , X. Tan , Acta Mater. 2017, 128, 337.

[20] R. Cheng , Z. Xu , R. Chu , J. Hao , J. Du , G. Li , J. Eur. Ceram. Soc. 2016, 36, 489.

[21] J. Chen , Y. Wang , Y. Zhang , Y. Yang , R. Jin , J. Eur. Ceram. Soc. 2017, 37, 2365.

[22] L. Zhang , H. Wang , D. Wang , M. Guo , X. Lou , D. Wang , Adv. Funct. Mater. 2020, 30, 2004641.

[23] L. Zhang , X. Ren , M. A. Carpenter , Phys. Rev. B 2017, 95, 054116.

[24] M. Acosta , N. Khakpash , T. Someya , N. Novak , W. Jo , H. Nagata , G. A. Rossetti , J. Rödel , Phys. Rev. B 2015, 91, 104108.

[25] L. Zhang , M. Zhang , L. Wang , C. Zhou , Z. Zhang , Y. Yao , L. Zhang , D. Xue , X. Lou , X. Ren , Appl. Phys. Lett. 2014, 105, 162908.

[26] Q. Lin , R. Ding , Q. Li , Y. Y. Tay , D. Wang , Y. Liu , Y. Huang , S. Li , J. Am. Ceram. Soc. 2016, 99, 2347.

[27] L. He , Y. Ji , S. Ren , L. Zhao , H. Luo , C. Liu , Y. Hao , L. Zhang , L. Zhang , X. Ren , Ceram. Int. 2020, 46, 3236.

[28] L. Zhang , X. Lou , D. Wang , Y. Zhou , Y. Yang , M. Kuball , M. A. Carpenter , X. Ren , Phys. Rev. Appl. 2017, 8, 054018.

[29] X. Fang , H. Wang , L. He , Y. Sun , J. Du , H. Luo , D. Wang , L. Zhang , D. Wang , ACS Appl. Mater. Interfaces 2024, 16, 11497. 38391180 10.1021/acsami.3c17262

[30] L. Zhang , L. Zhao , L. He , D. Wang , Y. Sun , D. Wang , X. Lou , L. Zhang , M. A. Carpenter , ACS Appl. Mater. Interfaces 2022, 14, 1434. 34978786 10.1021/acsami.1c19856

[31] J. Gao , X. Hu , L. Zhang , F. Li , L. Zhang , Y. Wang , Y. Hao , L. Zhong , X. Ren , Appl. Phys. Lett. 2014, 104, 252909.

[32] H. Tao , J. Wu , D. Xiao , J. Zhu , X. Wang , X. Lou , ACS Appl. Mater. Interfaces 2014, 6, 20358. 25384464 10.1021/am505887y

[33] C. Ma , H. Guo , S. P. Beckman , X. Tan , Phys. Rev. Lett. 2012, 109, 107602. 23005327 10.1103/PhysRevLett.109.107602

[34] C. Ma , X. Tan , Solid State Commun. 2010, 150, 1497.

