# Supplementary material for: Superior Electromechanical Power at Rare‐Earth Manipulated Glassy Morphotropic Phase Transitions
Source: Adv Sci (Weinh). 2025 Jun 4;12(27):2415533. doi: 10.1002/advs.202415533 (PMC12279196; doi:10.1002/advs.202415533)
Supplement: Supplementary file 1 — Supporting Information [file ADVS-12-2415533-s001.docx]

Supportive Information

**Title** Superior Electromechanical Power at Rare-earth Manipulated Glassy Morphotropic Phase Transitions

Le Zhang, Liqiang He, Shuyuan Xu, Cunle Bo, Dong Wang*, Haoyu Wang, Yunlong Sun, Kaiyun Chen*, Junkai Deng, Zibin Chen, Danyang Wang*，Michael A. Carpenter and Sen Yang*

**1.** **Experimental and Calculation details**

***Fabrication of polycrystalline ceramics*:** *x* mol% Sm-modified 94%(Bi_0.5_Na_0.5_)TiO_3_-6%BaTiO_3_-1%Mn( *x=*0.1, 0.2, 0.3, 0.4 in the manuscript, abbreviated as 1,2,3.4 Sm) ceramic specimens were synthesized employing conventional sintering procedures with the starting oxides of Bi_2_O_3_ (99.9%, *Sigma-Aldrich*), Na_2_CO_3_ (99.5%*, Sigma-Aldrich*), BaCO_3_ (99.98%*, Sigma-Aldrich*), TiO_2_ (99%*, Sigma-Aldrich*), Sm_2_O_3_ (99%*, Sigma-Aldrich*) and MnO_2_ (99%*, Sigma-Aldrich*). The calcining and sintering was carried out at 1200-1250 K for 2 h and 1350-1400 K for 4 h in air.

***Properties Characterization:*** The room-temperature crystal structures of the poled xSm ceramics were characterized by x-ray diffractometer (Bruker D8 advance) with an accelerating voltage of 40kV and a current of 40mA. The target used for X-ray diffraction is Cu target with a wavelength of 1.54056Å. The scanning speed and step length are set as 1 degree/min and 0.02 degree/step. The refinement for the XRD spectra was conducted using the Rietveld method via HighScore Plus software. The phase structure and percentage of xSm samples are obtained with a low Rwp of 6.4-7.9% accordingly. The temperature dependence of dielectric permittivity *ε*_r_ were obtained using an LCR meter (*Agilent, E4980 Precision Meter*) with a temperature-controlled sample stage (*Linkam, HFS600E-PB4*). The temperature dependent *P-E* and *S-E* loops were measured by a Radiant Multiferroic Ⅱ ferroelectric workstation connected with a temperature-controlled chamber (293K-373K). The temperature dependent elastic modulus curves are measured in dynamic modulus analysis device with a frequency range of 0.2-20Hz. Transmission electron microscopy (TEM) observations were carried out using a Themis Z double-corrected microscope with a high-angle annular dark-field (HAADF) detector.

**Statistical Analysis:** The sample size used for dielectric, ferroelectric, piezoelectric property determination and SEM characterization are 500-600μm in thickness and 6mm in diameter. The Software used for the experimental data analysis and presentation is Origin software.All the tests are repeated for 2-3 times to make sure the data repeatable and validity.

***Details and parameters of DFT simulations:*** Our DFT calculations were conducted by using the Vienna Ab- inito Simulation Package (VASP)^[1]^. The Perdew-Burke-Ernzerhof revised for solids (PBEsol) exchange-correlation functional^[2-3]^ and projector-augmented wave method^[4]^ was adopted with the valence-electron configurations of 5d^10^6s^2^6p^3^ for Bi, 2p^6^3s^1^ for Na, 3p^6^3d^2^4s^2^ for Ti, 2s^2^2p^4^ for O, 5s^2^5p^6^6s^2^ for Ba, and Sm_3 for Sm. The cut-off energy of 500 eV for all calculations. The $\sqrt{2} \times\sqrt{2} \times2$ super unit cell of the cubic (Na0.5Bi0.5)TiO3 was adopted, in which a^0^a^0^c^+^ and a^–^a^–^c^–^ rotation was induced for P4bm and R3c phase, respectively, and Ti distortion along c-axis but without rotation were introduced for P4mm phase. The atomic positions and lattice constants were fully relaxed until the total energy difference and forces were less than 10^−4^ eV and 0.01 eV/Å, respectively. The Gamma centre k-mesh of 2 × 2 × 2 was adopted for geometrical optimization calculations for all the above three^[5]^. The climbing image nudged elastic band (Cl-NEB) calculations^[6]^ were performed to estimate the energy barriers among P4bm, P4mm, and R3c with Ba, and Ba/Sm co-doped system, phase transitions. Periodic boundary condition was applied in all three directions.

***Details and parameters of phase field simulations:*** In phase field simulations, the total free energy *F* of the proposed system is given in the summation form of Landau free energy, *F_Landau_* ($\int f_{Landau}dV$), the elastic strain energy, *F*_elas_ ($\int f_{elas}dV$),the electrostatic energy, *F*_elec_($\int f_{elec}dV$), the gradient energy, *F*_grad_ ($\int f_{grad}dV$) and extra energy caused by local electric field from doping, *F_LEF_*($\int f_{LEF}dV$) , as described in Equation (1):

$F=F_{Landau}+F_{grad}+F_{elas}+F_{elec}+F_{LEF}$ (1)

$f_{Landau}=A_{1}\left( P_{1}^{2}+P_{2}^{2}+P_{3}^{2} \right)-A_{2}\left( P_{1}^{4}+P_{2}^{4}+P_{3}^{4} \right)+A_{12}\left( P_{1}^{2}P_{2}^{2}+P_{2}^{2}P_{3}^{2}+P_{1}^{2}P_{3}^{2} \right)+A_{13}\left( P_{1}^{4}P_{2}^{2}+P_{2}^{4}P_{3}^{2}+P_{1}^{4}P_{3}^{2}+P_{1}^{2}P_{2}^{4}+P_{2}^{2}P_{3}^{4}+P_{1}^{2}P_{3}^{4} \right){+A_{14}\left( P_{1}^{2}P_{2}^{2}P_{3}^{2} \right)+A}_{3}\left( P_{1}^{6}+P_{2}^{6}+P_{3}^{6} \right)$(2)

where *f*_Landau_ stands for the density of Landau free energy^[7-9]^ as expanded in Equations (2), in which the rotation barriers between different ferroelectric phases are controlled and depicted. The constants *A*_i_ (i =1-3), *A*_1j_ (j =2-4) in *f*_Landau_ are the Landau coefficients as a function of the *c* (doping level) and T (temperature). The strain heterogeneity induced by Sm dopant will be induced in the *F*_elas_ and while the polarization rotation dynamics of structural heterogeneity can be considered in the *F*_landau_ and *F*_LEF_. The detailed parameters used are given in the simulation section. The gradient energy density *f*_grad_ is given as $f_{grad}=\frac{1}{2}G_{11}(\sum_{i,j}^{3} (P_{i,j})^{2})$, where *G*_11_ is the gradient energy coefficient. The elastic energy density *f_elas_* is expressed by $f_{elas}=\frac{1}{2}C_{ijkl}e_{ij}e_{kl}=\frac{1}{2}C_{ijkl}(\varepsilon_{ij}-\varepsilon_{ij}^{0}+\varepsilon_{ij}^{local})(\varepsilon_{kl}-\varepsilon_{kl}^{0}+\varepsilon_{kl}^{local})$, where *C_ijkl_* is the elastic constants, *e_ij_* the elastic strains, *ε_ij_* the total strains, *ε_ij_^0^* the spontaneous strains and *ε_ij_^local^* the local strain from lattice distortions. The electrostatic energy density *f_elec_* is calculated by the equation $f_{elec}=\sum_{i=1,2,3} -\frac{1}{2}E_{i}P_{i}-\frac{1}{2}E_{i,depol}\overline{P_{i}}$, where *E*_i_ denotes the heterogeneity electric field due to the dipole-dipole interactions, *E*_i,depol_, the average depolarization field due to the surface charge, $\overline{P_{i}}$, the average polarization. The extra energy density caused by local electric field *f_LEF_* is written by $f_{LEF}=\sum_{i=1,2,3} -E_{i}^{L}P_{i}$. A set of random local electric field with different orientations (*E*_1_*^L^*, *E*_2_*^L^*, *E*_3_*^L^*, with Gaussian distribution $f(E_{i}^{L})=0.2\cdot e^{(-0.008(E_{i}^{L}-11)^{2})}$) have been used to describe the local field in our simulations^[10]^. The parameters used in the calculations are chosen according to references^[11-12]^. The chosen Landau coefficients could produce a relaxor – R phase(less T) with long range order upon cooling. The energy barrier decided by *A*_2_ shows a decrease first and then increase with the increase of doping level *c*.

**References**

[1] G. Kresse, J. Furthmüller, *Computational Materials Science* **1996**, 6, 15.

[2] J. P. Perdew, K. Burke, M. Ernzerhof, *Physical Review Letters* **1996**, 77, 3865.

[3] J. P. Perdew, A. Ruzsinszky, G. I. Csonka, O. A. Vydrov, G. E. Scuseria, L. A. Constantin, X. Zhou, K. Burke, *Physical Review Letters* **2008**, 100, 136406.

[4] P. E. Blochl, *Physical Review-Section B-Condensed Matter* **1994**, 50, 17953.

[5] H. J. Monkhorst, J. D. Pack, *Phys. Rev. B* **1976**, 13, 5188.

[6] G. Henkelman, B. P. Uberuaga, H. Jónsson, *The Journal of Chemical Physics* **2000**, 113, 9901.

[7] D. V. Karpinsky, E. A. Eliseev, F. Xue, M. V. Silibin, A. Franz, M. D. Glinchuk, I. O. Troyanchuk, S. A. Gavrilov, V. Gopalan, L.-Q. Chen, A. N. Morozovska, *npj Computational Materials* **2017**, 3, 20.

[8] A. J. Bell, L. E. Cross, *Ferroelectrics* **1984**, 59, 197.

[9] Y. L. Li, S. Y. Hu, Z. K. Liu, L. Q. Chen, *Applied Physics Letters* **2002**, 81, 427.

[10] D. Wang, X. Ke, Y. Wang, J. Gao, Y. Wang, L. Zhang, S. Yang, X. Ren, *Phys. Rev. B* **2012**, 86, 054120.

[11] T. Yang, X. Ke, Y. Wang, *Scientific Reports* **2016**, 6, 33392.

[12] X. Q. Ke, D. Wang, X. Ren, Y. Wang, *Phys. Rev. B* **2013**, 88, 214105.

1. Supportive Figures and Tables


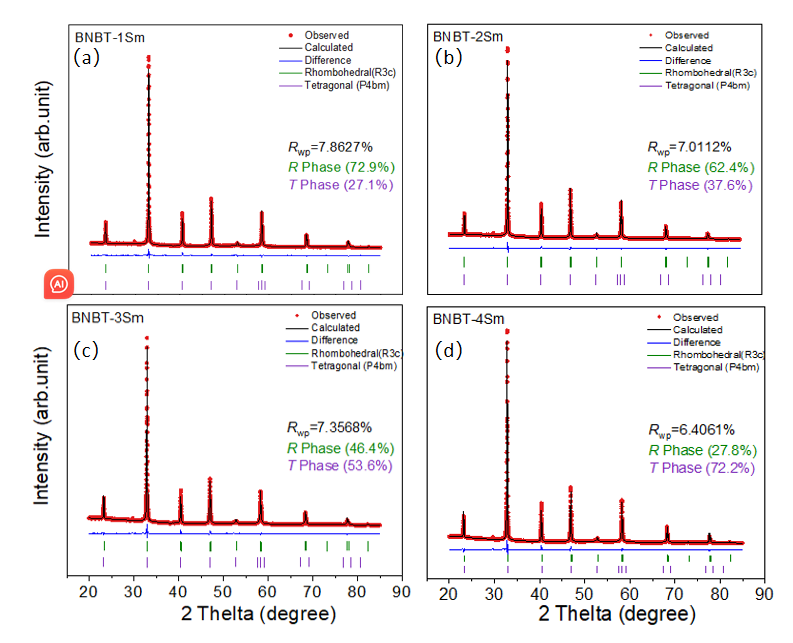


Fig.S1 The refinement results of 1-4Sm ceramic specimen. The results indicate that the increase of Sm dopants can stabilize the T phase with P4bm symmetry and hinder the formation of R3c.


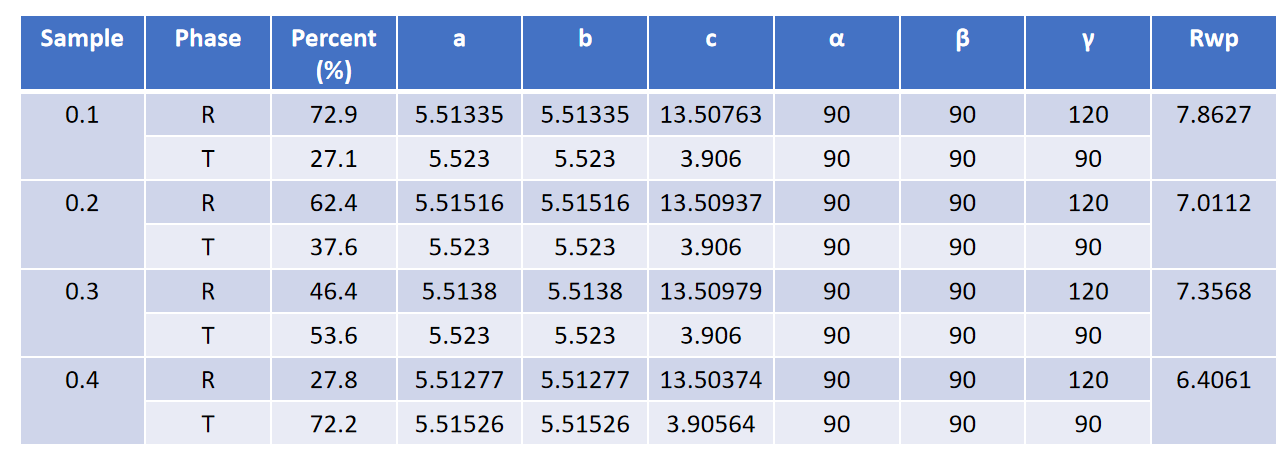


Table S1 The summary for the percentage, lattice parameters of R and T phase in XRD refinement results

**
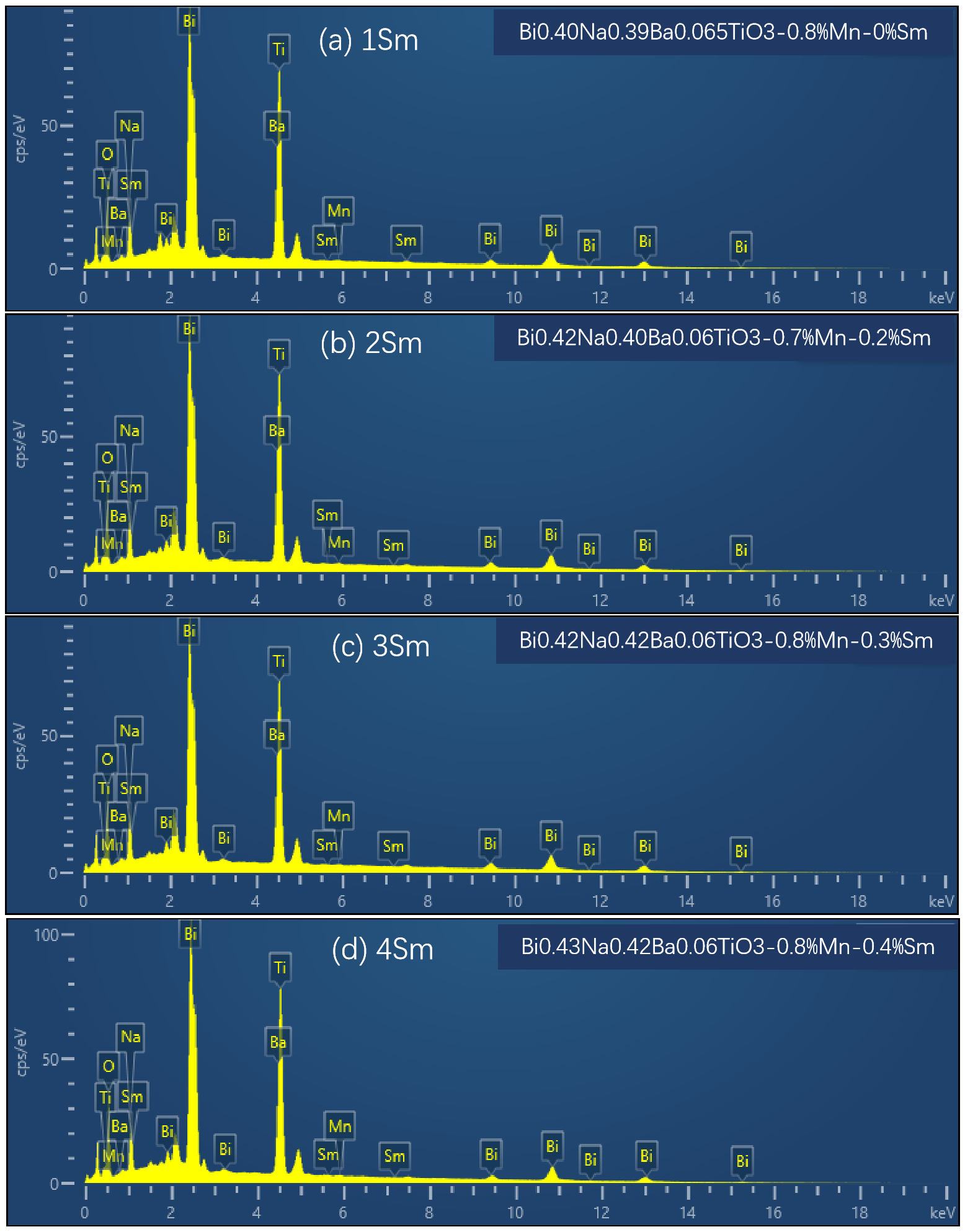
**

Figure S2 EDS analysis of 1-4Sm as-grown ceramic samples in SEM technique


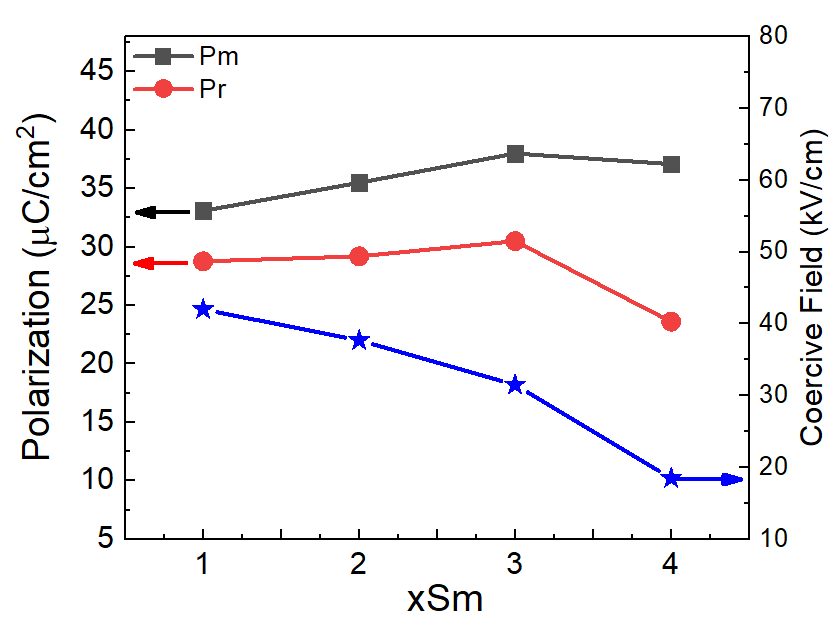


Figure S3 The variation of *Pm, Pr, Ec* with changing the Sm composition
